# Implications of incidental findings from lung screening for primary care: data from a UK pilot

**DOI:** 10.1038/s41533-021-00246-8

**Published:** 2021-06-07

**Authors:** Emily C. Bartlett, Jonathan Belsey, Jane Derbyshire, Katie Morris, Michelle Chen, James Addis, Maria Martins, Carole A. Ridge, Sujal R. Desai, Saeed Mirsadraee, Simon Padley, Sarah Whiteside, Pritti Vaghani, Jaymin B. Morjaria, Samuel V. Kemp, Anand Devaraj

**Affiliations:** 1 Royal Brompton and Harefield NHS Foundation Trust, Department of Radiology, London, UK; 2 National Heart and Lung Institute, Imperial College, London, UK; 3 JB Medical Ltd, Sudbury, UK; 4 RM Partners, the West London Cancer Alliance, London, UK; 5 Respiratory Biomedical Research Unit, Royal Brompton Hospital and Harefield NHS Foundation Trust, London, UK; 6 North End Medical Centre, London, UK; 7 Macmillan GP for Hillingdon CCG, New York, NY USA; 8 Royal Brompton and Harefield NHS Foundation Trust, Department of Respiratory Medicine, Harefield Hospital, Harefield, UK; 9 Royal Brompton and Harefield NHS Foundation Trust, Department of Respiratory Medicine, London, UK

**Keywords:** Population screening, Health care economics, Diagnosis

## Abstract

Regional lung cancer screening (LCS) is underway in England, involving a “lung health check” (LHC) and low-dose CT scan for those at high risk of cancer. Incidental findings from LHCs or CTs are usually referred to primary care. We describe the proportion of participants referred from the West London LCS pilot to primary care, the indications for referral, the number of general practitioner (GP) attendances and consequent changes to patient management, and provide an estimated cost-burden analysis for primary care. A small proportion (163/1542, 10.6%) of LHC attendees were referred to primary care, primarily for suspected undiagnosed chronic obstructive pulmonary disease (55/163, 33.7%) or for QRISK® (63/163, 38.7%) assessment. Ninety one of 159 (57.2%) participants consenting to follow-up attended GP appointments; costs incurred by primary care were estimated at £5.69/LHC participant. Patient management changes occurred in only 36/159 (22.6%) referred participants. LHCs result in a small increase to primary care workload provided a strict referral protocol is adhered to. Changes to patient management arising from incidental findings referrals are infrequent.

## Introduction

Targeted lung cancer screening (LCS) with CT is being introduced in the UK, via a number of pilot programmes. The model of lung screening that has been most widely used in the UK to date involves so-called “lung health checks” (LHCs)^1–4^, comprising a clinical consultation, spirometry, and a lung cancer risk score calculation. In these pilot programmes, the lung cancer risk score calculation is used to determine eligibility for a low-dose computed tomography (LDCT) scan.

It is known that LCS participants demonstrate high mortality rates from cardiovascular disease and non-lung cancer respiratory disease^5,6^, and over recent years there has been particular interest in using the LHC to identify those who may benefit from treatment for undiagnosed cardiovascular and respiratory disease^7–10^. The clinical consultation and spirometry within the LHC allow for the identification of wider health issues, such as potentially undiagnosed chronic obstructive pulmonary disease (COPD)^8,10^, while the identification of coronary calcification on LDCT can be used as a marker of cardiovascular risk to identify participants who may benefit from lipid-lowering therapy^7^. Consequently, there has been particular interest in using the LHC to identify those who may benefit from treatment for undiagnosed cardiovascular and respiratory disease.

Individuals with non-malignant incidental findings in targeted lung screening pilots are typically referred to primary care for further management. This includes both incidental findings identified at LDCT, as well as conditions such as suspected undiagnosed COPD identified as part of the LHC. However, the downstream consequences of identification of non-lung cancer conditions in the LHC model for primary care and screening participants have not been widely evaluated. Little is known about the impact of referrals on primary care workload, whether recommendations made by screening programmes are implemented, and whether there are subsequent changes to patient management. In addition, no prior work has evaluated the overall financial cost to primary care, resulting from referrals from LCS programmes.

A targeted LCS pilot study commenced in West London in August 2018, the baseline results of which have been previously published^11^. The aims of this sub-study were to evaluate the impact of identification of incidental findings from LHCs and targeted lung screening on primary care and screening participants. We report (1) the number and incidence of participants referred to primary care from the LHC for incidental finding management and the indications for referral, (2) the number and proportion of subsequent general practitioner (GP) attendances, (3) the incidence of changes in patient management, and (4) a cost-burden analysis for primary care based on the West London pilot.

## Results

### Overall attendance at a LHC and eligibility for LDCT

Of 8366 subjects invited for a LHC, 1542 attended a LHC appointment, had a clinical consultation and a risk score calculation^11^. Spirometry was performed on 1532/1542 participants (99.4%); 1145/1542 (74.2%) had a LDCT scan.

### Primary care referrals: numbers and indications

One hundred and sixty-three participants (163/1542, 10.6%) aged between 60 and 75 were referred to primary care, cumulatively for 169 indications. Of these, 159/163 (97.5%; 110 males, 49 females) consented to follow-up. Indications for primary care referral are shown in Table 1. Six of the 159 participants had two reasons for referral.

**Table 1:** Indications for primary care referral in 159 consenting participants from the West London screening pilot.

Indication for referral to primary care	Number of referrals to primary care (proportion attending a LHC, %)
Obstructive spirometry with chronic cough or dyspnoea; no known diagnosis of chronic obstructive pulmonary disease (COPD) or asthma	55 (55/1542, 3.6%)
Moderate or severe coronary artery calcification on LDCT; not already taking lipid-lowering therapy and without known ischaemic heart disease	63 (63/1542, 4.1%)
Moderate or severe aortic valve calcification on LDCT	18 (18/1542, 1.2%)
Aortic aneurysms (referred only to primary care if not urgent/significantly enlarged)	4 (4/1542, 0.3%)
Severely dilated pulmonary artery suggestive of pulmonary hypertension	1 (1/1542, 0.06%)
Other imaging finding requiring further primary care investigation or referral	
•Liver, adrenal, or renal lesions	4 (4/1542, 0.3%)
•Suspected pneumonia	1 (1/1542, 0.06%)
•Miscellaneous musculoskeletal findings	2 (2/1542, 0.1%)
Red flag symptoms	17 (17/1542, 1.1%)
Total number of referrals	165 (in 159 participants)

### Primary care attendances

**Fig. 1:**
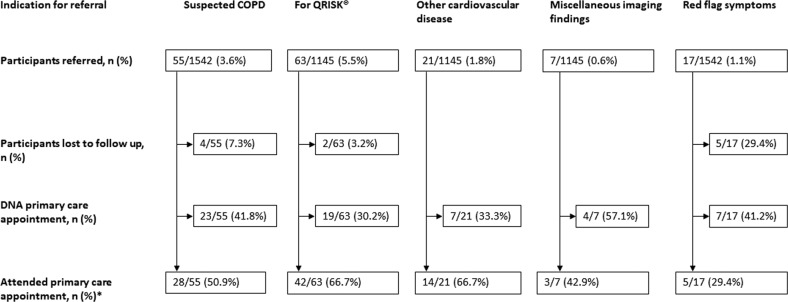
Flow charts demonstrating attendance of participants referred to primary care for incidental imaging findings or further clinical assessment following a LHC. For participants referred, the denominator is the number of participants having a LHC (where 1542) or the number of participants having a LDCT scan (where 1145).

Primary care attendances are summarised in Fig. 1.

Attendance at primary care appointments for red flag symptoms was lower than for other referral indications. Among those referred for red flag symptoms who did not attend GP appointments (7/17, 41.2%), two participants denied previously reporting the red flag symptom, and four participants considered the symptom not of significance and had not attended a GP appointment. One participant sought private medical care, rather than visiting their GP. In total, 91 participants attended primary care appointments (2 participants for two indications).

### GP investigations and consequent changes in patient management

Of the twenty-eight participants attending a GP appointment for suspected COPD, twenty-one participants (21/55 originally referred, 38.2%) had been referred onward for a community respiratory assessment for COPD. Sixteen participants (16/55, 29.1%) received a new respiratory diagnosis: either COPD (14/55, 25.5%) or asthma (2/55, 3.6%). Of these, pharmacotherapy was commenced in 6/16 (37.5%), and 1/16 (6.3%) began pulmonary rehabilitation. Actual changes in management therefore occurred for 7/55 (12.7%) referred.

Of the forty-two participants (42/63 originally referred, 66.7%) with moderate or severe coronary calcification on LDCT not already taking statin therapy and reviewed in primary care for a QRISK assessment^12,13^, 1 was already taking lipid-lowering therapy, previously unreported at the LHC. Thirty of the remaining 41 (73.2%) participants had a QRISK® calculation (all QRISK® scores > 10%). Twenty four of 41 (58.5%) participants started statin therapy. One participant commenced statin therapy following referral to a rapid access chest pain clinic, independent of the LHC recommendation. Sixteen participants (16/63, 25.4%) declined statin therapy. Therefore, a total of 24/63 (38.1%) participants referred for a QRISK® had a change in management, as a result of the LHC.

Among the 21 participants referred (for 23 different indications) for other suspected cardiovascular disease on LDCT, echocardiograms were performed in 8/18 (44.4%) participants for whom echocardiograms were recommended; one of these participants was subsequently referred to cardiology because of critical aortic stenosis and underwent aortic surgery. Among these 21 participants, 3 non-urgent secondary care referrals were recommended in participants with moderate aortic dilatation (*n* = 2) and heavy aortic valve calcification (*n* = 1); 1/3 (33.3%) had an echocardiogram rather than a cardiology referral, and 2/3 (66.7%) were referred to secondary care (cardiac surgery). Four of 21 (19.0%) participants remain under surveillance in secondary care.

Of 81 participants for whom a QRISK® calculation or echocardiogram was recommended, 6/81 (7.4%) were referred for cardiology review following primary care consultations, although this had not been specifically recommended by the LHC team.

Of the 3/7 (42.9%) participants reviewed in primary care for miscellaneous imaging findings, one (14.3%) was subsequently referred to secondary care (urology), and two participants (2/7, 28.6%) were managed within primary care, only 1 of whom had further investigations (blood tests). No malignancies were detected in the three, who attended GP appointments.

Among the 5/17 (29.4%) participants with red flag symptoms without lung cancer who were reviewed in primary care, 1 was referred for a colonoscopy and breast screen. The remaining four had no further investigations. None of the participants reviewed were diagnosed with cancer.

Therefore, in total 36/159 (22.6%) of those undergoing a LHC had a change in management.

### Primary care cost-burden analysis

The cost of 91 GP appointments was £3003.00. Twenty-one participants attended community COPD reviews, costing £2919.00. Subsequent secondary care referrals and costs for further clinical or imaging investigations cost £2854.00 (Table 2). The total cost of primary care investigations and referrals resulting from LHC recommendations was £8776.00. This was at a mean cost of £55.19/consenting patient referred to primary care, or £5.69/patient attending a LHC.

**Table 2:** Costs of subsequent imaging studies and secondary care referrals following LHC recommendations.

Activity or investigation	Cost/unit	Number performed	Total cost
Direct access echocardiogram	£64.00	9	£576.00
Cardiology referral	£157.00	7	£1099.00
Outpatient colonoscopy	£406.00	1	£406.00
Urology referral	£142.00	1	£142.00
Cardiac surgery referral	£296.00	2	£592.00
Mammogram	£39.00	1	£39.00
			£2854.00

## Discussion

In the West London lung screening pilot, a strict protocol was adhered to for referrals to primary care for non-lung cancer findings. A numerically small proportion (163/1542, 10.6%) of participants attending a LHC were referred to primary care for further management or investigation. The majority (141/165, 85.4%) of indications for referral to primary care were either cardiovascular or respiratory, these occurring in 8.8% (135/1542) of participants undergoing a LHC. Of those consenting to follow-up, 91/159 (57.2%) attended GP appointments, and costs incurred by primary care were overall estimated at £5.69/patient attending a LHC. Changes in patient management directly initiated in primary care or following subsequent secondary care referral were uncommon, occurring in only 36/159 (22.6%) referred participants consenting to follow up, or 36/1542 (2.3%) of those undergoing a LHC. These most frequently occurred following recommendations for QRISK® score evaluation, following the identification of moderate or severe coronary artery calcification on CT (24/1542, 1.6%), and less frequently in those referred with suspected COPD based on obstructive spirometry with symptoms (7/1542, 0.5%) or in patients with other cardiovascular indications for review (5/1542, 0.3%).

Two recent UK-based lung screening studies using a LHC model have reported cardiovascular QRISK® scores and prevalence of coronary artery calcification in the screening population^7,9^. In the Lung Screening Uptake Trial, 29% of those undergoing CT screening had moderate or severe coronary calcification^7,14^. In the Manchester lung screening pilot, almost 25% of participants eligible for primary prevention therapy according to QRISK®, not already taking a statin, had moderate or severe coronary calcification on LDCT^9^. Importantly, neither study evaluated GP attendances following LHCs nor impact on patient management, both of which are crucial for increasing the cost-effectiveness of screening programmes in a real-world setting. A strength of this study is that follow-up data were available for the majority (148/163, 90.8%) of participants.

Previous studies have also sought to quantify the proportion of attendees at screening programmes with undiagnosed COPD and found 18.6–38.2% of lung screening participants attending a LHC have obstructive spirometry without a previous diagnosis of COPD^3,8,10^. However, data on patient outcomes as a result of identifying undiagnosed COPD in screening are lacking. In the West London pilot, only 55/1542 (3.6%) lung screening attendees were referred to primary care. Our referral rate was lower than other pilots as a formal referral for COPD assessment was made only in symptomatic participants not known to have asthma or COPD in line with national and international guidelines^15,16^. Consequently, of those undergoing a LHC, only 16/1542 (1.1%) received a new respiratory diagnosis (comprising 29.1% of those referred for a community respiratory/COPD review), reflecting relatively low GP attendance, as well as the fact that a small number of participants already had a diagnosis of COPD within their primary care records, of which they were unaware.

Our findings of only 57.2% of referred participants actually attending primary care appointments was unexpected, given that participants had opted-in to the pilot, indicating some interest in personal health. Participants may not have understood the purpose of the referral, or had difficulty in obtaining a GP appointment, reasons cited elsewhere for not attending NHS health checks^17^. In some instances, participants made a deliberate choice not to attend, possibly because they may not have considered the indication for referral to be a serious health problem, or urgent matter. On the part of GPs, this may reflect lack of engagement with the recommendations in letters, the clinical judgement of the GPs, or time constraints. Further work would be informative, to maximise the potential benefits from LHC programmes.

This study also aimed to quantify the downstream costs to primary care of identifying incidental findings in LCS using LHCs, an area which has not been studied previously. We found that overall costs/screening participant attending a LHC were very low (£5.69/participant). This study was not intended as a cost-effectiveness analysis (rather a cost-burden analysis) given the complexities of quantifying cost-benefits for several interventions in a small number of participants. It can reasonably be assumed that the costs described may be offset by benefits obtained through diagnosis and health interventions. The extent of this benefit is however currently unknown.

Costs to primary care may have been underestimated in our study for three reasons. Firstly, for some incidental findings we made direct referrals to secondary care, which we did not incorporate in our cost analysis. However, it is possible that some of the referrals may be managed by primary care. Secondly, a single GP appointment was assumed. However, the proportion of participants who might attend multiple GP appointments is thought to be small. Thirdly, data were not collected on further investigations that might result from management changes, including serial laboratory tests (such as lipid profile), which would marginally increase costs.

In summary, data from the West London pilot indicate that targeted LCS results in a small but inevitable increase to primary care workload, but that referrals to primary care can be kept to a reasonable level and at low cost provided there is adherence to a strict protocol. As screening is rolled-out in similar pilots nationally, data from other similar studies will be required to validate our findings. Identifying incidental findings from a LHC and screening CT has the potential to improve patient health, in particular relating to lowering cardiovascular risk and treating undiagnosed COPD. However, these benefits cannot be assumed, as we have shown in our study that impact on patient management may be limited. Reasons for this are unclear and warrant further investigation.

## Methods

### Study population and protocol

A total of 8366 smokers and ex-smokers aged 60–75 from 17 general practices in West London were invited for a LHC between August 2018 and April 2019. Lung cancer risk was assessed by two validated risk models^18,19^. A LDCT was offered to participants meeting a LLP_v2_ 5-year lung cancer risk threshold of ≥2.0% and/or a PLCO_M2012_ 6-year risk threshold of ≥1.51%^18,19^.

A project steering group, including primary and secondary care representatives, agreed a protocol for participant referral to primary care for incidental findings at the outset of the pilot. Table 3 documents the indications for referrals to primary care, and recommendations made in different clinical scenarios. Participants were referred either due to reported clinical symptoms and/or abnormal spirometry during the LHC appointment, or as a result of specific incidental findings detected on LDCT, if performed. In line with national NHS England guidance, participants were only referred to primary care for incidental findings suspicious for a clinically significant underlying pathology (e.g., aortic aneurysms), or where there was a possible primary care intervention which might alter patient prognosis (e.g., a QRISK® score with a view to lipid-lowering therapy)^20–23^.

**Table 3:** Indications for referral to primary care and recommended primary care actions.

LHC clinical scenario or LDCT incidental findings	Recommended primary care action
Obstructive spirometry with chronic cough or dyspnoea; no known diagnosis of COPD or asthma	COPD review
Moderate or severe coronary artery calcification on LDCT; not already taking lipid-lowering therapy, and not known to have a history of ischaemic heart disease	QRISK® cardiovascular risk assessment score^12,13^
Moderate or severe aortic valve calcification, or evidence of other cardiac valve disease	Echocardiogram referral
Aortic aneurysms (referred only via primary care if nonurgent)	Vascular or cardiothoracic referral, and/or echocardiogram referral
Dilated pulmonary artery suggestive of pulmonary hypertension	Echocardiogram referral
Other imaging finding requiring further primary care investigation or referral •Suspicious liver, adrenal, or renal lesions •Suspected pneumonia •Miscellaneous musculoskeletal findings	Referrals as specified by radiologist: •Imaging or biochemical tests or onward referral •For clinical assessment •Recommendations for investigation at discretion of reporting radiologist

In addition participants reporting “red flag” symptoms at the LHC (excluding those in whom a lung cancer diagnosis was made) were referred for primary care assessment; namely participants with red flag symptoms^24^, not specific to lung cancer, but possibly indicating an alternative cancer diagnosis (weight loss, fatigue, or chest pain). GPs were informed of symptoms, but not given specific recommendations for further investigation. Results letters and recommendations were sent to participants and their GPs within 2 weeks of the LHC appointment.

### Ethics considerations

Follow-up of participants in primary care was performed as a service evaluation project and was registered accordingly at the Royal Brompton Hospital. As a service evaluation, and in line with national guidance^25^, this study did not require Health Research Authority approval. Overall governance of this project was provided by the West London screening pilot steering group, comprising representatives from primary and secondary care. Due consideration was given to ethical and data protection requirements. In line with the Healthcare Quality Improvement Partnership guidelines on service evaluation^26^, the steering group deemed that due consideration had been given to consent of participants, and that written consent was not required for this service evaluation project. Verbal consent for patient and GP follow-up was obtained from all participants reported in this study. This was established at the time of the LHC and was recorded electronically in a custom-built secure project database used for all LHC data collection. Non-consenters were excluded from analysis.

### Data collection and analysis

Primary care records were reviewed by a member of the study team in conjunction with primary care staff 3–9 months after the LHC. Where specific recommendations had been made to primary care the following data were collected:

(i) Consequent patient attendance rate in primary care,
(ii) Implementation rate of recommendations for investigations,
(iii) Percentage of participants with changes to management following investigations (including lung rehabilitation or pharmacotherapy),
(iv) Subsequent secondary care referrals made by GPs either directly as recommended, or independently by the GP.

In some instances, it was found that participants were referred to primary care based on inaccurate information given by participants during the LHC. For example, some participants were unaware that they were taking lipid-lowering therapy, or that they already had a diagnosis of COPD. In these cases, this became apparent upon review of GP records at the time of data collection.

In addition, data were collected (GP attendances and investigations performed) for participants who had been referred to GPs for red flag symptoms. Those who died or moved out of area in the period between the LHC appointment and the time of follow-up were considered “lost to follow up”. Categorical data are reported as proportions and percentages.

### Primary care costs

UK-based primary care costs for subsequent GP appointments and investigations were estimated from published sources, including the Personal Social Services Research Unit (PSSRU)^27^, and NHS England Tariffs (2018/2019)^28^, and Reference Costs (2017/2018)^29^ (Table 4). In the absence of a specific tariff for a community COPD review, where this occurred, the cost incurred by the GP was based on the cost of Respiratory Physiology—Full Lung Function testing^29^. When participants did visit their GP, a single GP appointment was assumed.

**Table 4:** Cost inputs and sources for primary care costs.

Activity or investigation	Cost/unit	Reference
GP appointment	£33.00	PSSRU. Unit costs of health and social care 2019^27^
Direct access echocardiogram	£64.00	NHS England. National Tariff 2018/19 [NHS Improvement]^28^
Cardiology referral	£157.00	NHS England. National Tariff 2018/19 [NHS Improvement]^28^
Community respiratory assessment (Respiratory Physiology—Full Lung Function testing)	£139.00	NHS England. NHS Reference Costs 2017/18 [NHS Improvement]^29^
Outpatient colonoscopy	£406.00	NHS England. National Tariff 2018/19 [NHS Improvement]^28^
Urology referral	£142.00	NHS England. National Tariff 2018/19 [NHS Improvement]^28^
Cardiac surgery referral	£296.00	NHS England. National Tariff 2018/19 [NHS Improvement]^28^
Mammogram (costed as Direct Access Plain Film)	£39.00	NHS England. NHS Reference Costs 2017/18 [NHS Improvement]^29^

When GPs referred to secondary care or organised investigations, costs are included, even if the advice recommended within the results letter was not followed exactly, to reflect real-world practice. The cost of subsequent investigations performed at the discretion of secondary care following the initial referral are not included in this analysis, which focuses instead on initial costs that can be attributed to a specific primary care practitioner.

### Reporting summary

Further information on research design is available in the Nature Research Reporting Summary linked to this article.

## Supplementary information

Reporting Summary

## Data Availability

The datasets used and analysed in the current study are available from the corresponding author on reasonable request. The approach taken for the study is detailed in the main text and could be reproduced in any similar screening pilot.
