# Supplementary material for: Implications of incidental findings from lung screening for primary care: data from a UK pilot
Source: NPJ Prim Care Respir Med. 2021 Jun 7;31:36. doi: 10.1038/s41533-021-00246-8 (PMC8184811; doi:10.1038/s41533-021-00246-8)
Supplement: Supplementary file 1 — Reporting Summary [file 41533_2021_246_MOESM1_ESM.pdf]

nature research

Corresponding author(s): Professor Anand Devaraj

Last updated by author(s): Apr 14, 2021

Reporting Summary

Nature Research wishes to improve the reproducibility of the work that we publish. This form provides structure for consistency and transparency in reporting. For further information on Nature Research policies, see our Editorial Policies and the Editorial Policy Checklist.

Statistics

For all statistical analyses, confirm that the following items are present in the figure legend, table legend, main text, or Methods section.

n/a	Confirmed	
☒	☐	The exact sample size (*n*) for each experimental group/condition, given as a discrete number and unit of measurement
☒	☐	A statement on whether measurements were taken from distinct samples or whether the same sample was measured repeatedly
☒	☐	The statistical test(s) used AND whether they are one- or two-sided<br>*Only common tests should be described solely by name; describe more complex techniques in the Methods section.*
☒	☐	A description of all covariates tested
☒	☐	A description of any assumptions or corrections, such as tests of normality and adjustment for multiple comparisons
☒	☐	A full description of the statistical parameters including central tendency (e.g. means) or other basic estimates (e.g. regression coefficient) AND variation (e.g. standard deviation) or associated estimates of uncertainty (e.g. confidence intervals)
☒	☐	For null hypothesis testing, the test statistic (e.g. *F*, *t*, *r*) with confidence intervals, effect sizes, degrees of freedom and *P* value noted<br>*Give P values as exact values whenever suitable.*
☒	☐	For Bayesian analysis, information on the choice of priors and Markov chain Monte Carlo settings
☒	☐	For hierarchical and complex designs, identification of the appropriate level for tests and full reporting of outcomes
☒	☐	Estimates of effect sizes (e.g. Cohen's *d*, Pearson's *r*), indicating how they were calculated

Our web collection on statistics for biologists contains articles on many of the points above.

Software and code

Policy information about availability of computer code

Data collection	No software was used
Data analysis	No software was used

For manuscripts utilizing custom algorithms or software that are central to the research but not yet described in published literature, software must be made available to editors and reviewers. We strongly encourage code deposition in a community repository (e.g. GitHub). See the Nature Research guidelines for submitting code & software for further information.

Data

Policy information about availability of data

All manuscripts must include a data availability statement. This statement should provide the following information, where applicable:

- Accession codes, unique identifiers, or web links for publicly available datasets
- A list of figures that have associated raw data
- A description of any restrictions on data availability

The datasets used and analysed in the current study are available from the corresponding author on reasonable request.

Field-specific reporting

Please select the one below that is the best fit for your research. If you are not sure, read the appropriate sections before making your selection.

☒ Life sciences ☐ Behavioural & social sciences ☐ Ecological, evolutionary & environmental sciences

For a reference copy of the document with all sections, see nature.com/documents/nr-reporting-summary-flat.pdf

Life sciences study design

All studies must disclose on these points even when the disclosure is negative.

Sample size	Individuals included in this service evaluation were those participants (159 consenting participants in total) from the West London screening pilot who had incidental CT imaging or clinical findings requiring subsequent review in primary care. All reported participants gave consent for GP follow up.
Data exclusions	Four participants who did not give consent for follow up were excluded from this service evaluation.
Replication	The findings were not replicated as this was a service evaluation of a lung cancer screening pilot. The methods are described and might easily be reproduced in a similar screening pilot.
Randomization	No randomization
Blinding	No blinding

Reporting for specific materials, systems and methods

We require information from authors about some types of materials, experimental systems and methods used in many studies. Here, indicate whether each material, system or method listed is relevant to your study. If you are not sure if a list item applies to your research, read the appropriate section before selecting a response.

Materials & experimental systems

n/a	Involved in the study	
☒	☐	Antibodies
☒	☐	Eukaryotic cell lines
☒	☐	Palaeontology and archaeology
☒	☐	Animals and other organisms
☒	☐	Human research participants
☐	☒	Clinical data
☒	☐	Dual use research of concern

Methods

n/a	Involved in the study	
☒	☐	ChIP-seq
☒	☐	Flow cytometry
☒	☐	MRI-based neuroimaging

Clinical data

Policy information about clinical studies

All manuscripts should comply with the ICMJE guidelines for publication of clinical research and a completed CONSORT checklist must be included with all submissions.

Clinical trial registration	NA
Study protocol	According to national guidelines, this study was a service evaluation. The protocol for the service evaluation is described within the text of this paper and was approved by the West London screening pilot steering group. The protocol for the larger West London screening pilot from which participants for this service evaluation were derived, including how participants were recruited to the screening pilot, are reported in the main results of the pilot study in Lung Cancer journal and available from the following DOI: 10.1016/j.lungcan.2020.07.027
Data collection	Participant data for the West London screening pilot was collated on an internal custom-built secure database within the Royal Brompton Hospital. This database was used to identify screening participants who required follow up in primary care, and who had also consented to GP follow up. Data from primary care was obtained between August 2018 and April 2019, 3-9 months after the participants involved had undergone a lung screening health check +/- a CT scan within the West London lung cancer screening pilot.
Outcomes	Outcome measures were as follows: 1) Number of participants referred to primary care from the West London screening pilot (as a proportion of those attending screening) 2) Subsequent participant attendance in primary care (as a proportion of those participants referred to primary care) 3) Patient management changes resulting from referrals to primary care from the West London screening pilot 4) Costs to primary care as a result of referrals from the West London screening pilot.
